# AAPM‐RSS Medical Physics Practice Guideline 9.b: SRS‐SBRT

**DOI:** 10.1002/acm2.14624

**Published:** 2025-03-12

**Authors:** Eileen Cirino, Stanley H. Benedict, Pamela J. Dupre, Per H. Halvorsen, Grace Gwe‐Ya Kim, Meral L. Reyhan, Christopher W. Schneider, Lei Wang, Carl P. Weaver, Sua Yoo

**Affiliations:** ^1^ Beth Israel‐Lahey Health Lahey Hospital and Medical Center Burlington Massachusetts USA; ^2^ Department of Radiation Oncology UC Davis Cancer Center Sacramento California USA; ^3^ Inova Health System Springfield Virginia USA; ^4^ Varian Advanced Oncology Solutions Palo Alto California USA; ^5^ Radiation Medicine and Applied Science University of California San Diego, La Jolla California USA; ^6^ Department of Radiation Oncology Rutgers Cancer Institute of New Jersey New Brunswick New Jersey USA; ^7^ Mary Bird Perkins Cancer Center Baton Rouge Louisiana USA; ^8^ Department of Radiation Oncology Stanford University Stanford California USA; ^9^ Radiation Oncology Piedmont Hospital Atlanta Georgia USA; ^10^ Radiation Oncology Duke University Medical Center Durham North Carolina USA

**Keywords:** QA, SBRT, SRS

## Abstract

The purpose of this Medical Physics Practice Guideline (MPPG) is to describe the minimum level of medical physics support deemed prudent for the practice of linear‐accelerator, photon‐based (linac) stereotactic radiosurgery (SRS), and stereotactic body radiation therapy (SBRT) services. This report is an update of MPPG 9.a^1^ published in 2017. As SRS and SBRT services are rapidly adopted into the community‐practice setting, this guideline has been developed to build on the work presented in MPPG 9.a and provide current appropriate minimum practice guidelines for such services.

## 1 ABOUT MEDICAL PHYSICS PRACTICE GUIDELINES

The American Association of Physicists in Medicine (AAPM) is a nonprofit professional society whose mission is “Advancing medicine through excellence in the science, education and professional practice of medical physics.” The AAPM has roughly 10 000 members and is the principal organization of medical physicists in the United States.

The AAPM will periodically define new practice guidelines for medical physics practice to help advance the science of medical physics and to improve the quality of service to patients throughout the United States. Existing Medical Physics Practice Guidelines (MPPG) will be reviewed for the purpose of revision or renewal, as appropriate, on their fifth anniversary or sooner.

Each MPPG represents a policy statement by the AAPM, has undergone a thorough consensus process in which it has been subjected to extensive review, and requires the approval of the Professional Council. The MPPG recognizes that the safe and effective use of diagnostic and therapeutic radiology requires specific training, skills, and techniques, as described in each document. Reproduction or modification of the published practice guidelines and technical standards by those entities not providing these services is not authorized.

The following terms are used in the AAPM practice guidelines:

1. Must and Must Not: Used to indicate that adherence to the recommendation is considered necessary to conform to this practice guideline. **While must is the term to be used in the guidelines, if an entity that adopts the guideline has shall as the preferred term, the AAPM considers that must and shall have the same meaning**.
2. Should and Should Not: Used to indicate a prudent practice to which exceptions may occasionally be made in appropriate circumstances.

## 2 INTRODUCTION

The purpose of this MPPG is to describe the minimum level of medical physics support deemed prudent for the practice of linear‐accelerator, photon‐based (linac) SRS, and SBRT services. This report is an update of MPPG 9.a^1^ published in 2017. As SRS and SBRT services are rapidly adopted into the community‐practice setting, this guideline has been developed to build on the work presented in MPPG 9.a and provide current appropriate minimum practice guidelines for such services.

### 2.1 Scope

This MPPG's scope includes medical physics support for the entire treatment process including acceptance testing, commissioning, technical process development, treatment planning and delivery, and quality assurance related to linac‐based SRS and SBRT, hereafter referred to as SRS‐SBRT. For ring‐mounted linac delivery systems, this document applies to SBRT only^1^ It also provides an introduction to magnetic resonance imaging‐guided linac delivery systems with the intention of further developing this topic in subsequent revisions of this guideline. This MPPG is not intended to address SRS‐SBRT procedures based on gamma‐ray and particle beam (proton or heavier) delivery systems.

### 2.2 Potential limitations and precautions

This MPPG describes the minimum level of medical physics support the AAPM and the Radiosurgery Society (RSS) deem prudent for the aforementioned scope. This document does not constitute a policy & procedure or SOP for a specific clinic—that is the professional responsibility of the clinic's Qualified Medical Physicist (QMP)^2^ through an active collaboration with the clinic's Medical Director and other clinical team members.

For this guideline, the terms quality management (QM), quality assurance (QA), and quality control (QC), as defined in a white paper by Amurao et al.^3^ and described in AAPM TG100 are used.^4^

1. Quality management (QM): An overall management system that includes establishing quality policies and quality objectives.
2. Quality assurance (QA): A component of QM that focuses on providing confidence that quality requirements will be fulfilled.
3. Quality control (QC): A component of QM focused on the fulfillment of quality requirements including the specific tests required.

SRS‐SBRT refers to both SRS and SBRT as currently defined by the ACR Practice Parameters. Stereotactic guidance includes current and evolving technologies such as image guidance, surface guidance, motion management, and enhanced immobilization utilized to support ≤1 mm targeting accuracy.

1. Stereotactic radiosurgery (SRS)—as defined in the American College of Radiology‐ the American Radium Society collaborative document: ACR‐ARS Practice Parameter for the Performance of Brain Stereotactic Radiosurgery^5^: “For the purpose of this document, SRS is strictly defined as radiation therapy delivered via stereotactic guidance with approximately 1 mm targeting accuracy to intracranial targets in 1 to 5 fractions.”
2. Stereotactic Body Radiation Therapy (SBRT)—as defined in the American College of Radiology—American Society for Radiation Oncology collaborative document: ACR‐ASTRO Practice Parameter for the Performance of Stereotactic Body Radiation Therapy^6^: “Although treatment of intracranial sites may be understood conceptually as a form of SBRT, for the purpose of this document, SBRT is strictly defined as radiation therapy delivered via stereotactic guidance with high levels of targeting accuracy to extracranial targets.”

This guideline is not a substitute for risk analysis as described in TG‐100^4^ as each practice environment is unique. Therefore, it is recommended that each institution review the TG100 report^4^ and perform an in‐depth analysis of their treatment processes prior to implementing SRS‐SBRT and prior to any implementation of new procedures or technology. Guidance for the risk analysis process can be found on the Workgroup for the Implementation of TG100 repository https://mpec.aapm.org/repository/home.php and practical suggestion guide https://www.aapm.org/QualitySafety/TG100/documents/PracticalSuggestionsTG100.pdf, (*see* *Appendix A* *for a path to sample FMEA documents*).

### 2.3 Summary of changes

This document continues the general outline of its predecessor document. The following is a summary of the changes:

1. Reference updates are included for all sections.
2. Terminology updates and clarification are included as deemed relevant by the authors for consistency. This includes a change in the definitions of tolerance and action levels to maintain consistent terminology with MPPG 8.b^7^ where applicable.
3. There is a modification in the language for QMP supervision of SRS‐SBRT. The new language requires personal supervision for the first treatment session of SRS‐SBRT and direct treatment supervision after the initial fraction. See the last bullet items in section III.B.2.i and note 2 in that section.
4. Small field measurement is no longer in a separate section. It is included in section V.B.1.a. on treatment delivery system commissioning.
5. Section V.B.1.d on Ancillary Systems now includes imaging systems.
6. Section VI has 5 tables and several edits that should be reviewed. The order of the tests in each table is changed from MPPG 9.a to more closely follow the order of tests in more recent publications including MPPG 8.b: dosimetric, mechanical, safety, end to end. Table 4 for Halcyon and Ethos is new. Table 5 for non‐radiographic localization systems is added for reader reference due to the increased use of this modality in SRS‐SBRT treatment systems.
7. Several sections have added comments and references for single‐isocenter multi‐target (SIMT) QA.

## 3 STAFF QUALIFICATIONS AND RESPONSIBILITIES

Each member of the SRS‐SBRT team must be appropriately trained, and each team member's responsibilities in the SRS‐SBRT process must be clearly defined to ensure a consistently safe and accurate treatment delivery (training is addressed in more detail in Section V.B.2.b of this document).

### 3.1 Supervision level

1. This document follows supervision levels defined in AAPM Policy AP‐125_A.^8^ For the delivery of all radiation therapy services, the two responsible professionals are the radiation oncologist and the QMP. All other team members work under the supervision of these professionals; clinical procedures are supervised by the radiation oncologist, and technical procedures are supervised by the QMP.
2. General Supervision: The procedure is performed under a QMP's overall direction and control. The QMP's presence is not required during the performance of the procedure, but must be available to communicate by phone to provide assistance and direction if needed. Under General Supervision, the training of the personnel who actually perform the procedure and the maintenance of the necessary equipment and supplies are the responsibility of the QMP.
3. Direct Supervision: A QMP must exercise General Supervision and be present in the facility and immediately available to furnish assistance and direction throughout the performance of the procedure. Direct Supervision does not require that the QMP be present in the procedure room or control room when the procedure is being performed.
4. Personal Supervision: A QMP must exercise General Supervision and be present in the procedure room or control room during the performance of the procedure.

### 3.2 Medical physicist

#### 3.2.1 Qualifications

The medical physicist with responsibility for the SRS‐SBRT program must meet the AAPM definition of a QMP^2^ for therapeutic medical physics. Appropriately trained medical physicists who do not meet the definition of a QMP must work under the supervision of a QMP. All medical physicists supporting the SRS‐SBRT program must have specific training in SRS‐SBRT prior to supervising patient‐specific procedures, including review of planning and treatment delivery procedures, equipment‐specific QA, and patient‐specific QA.

#### 3.2.2 Responsibilities

The role of the QMP is to assure the safe and effective use of radiation in medicine by performing or overseeing the scientific and technical aspects of procedures necessary to achieve this objective.^9^

The ACR‐ARS Practice Parameter for the Performance of Brain Stereotactic Radiosurgery,^5^ the ACR‐ASTRO Practice Parameter for the Performance of Stereotactic Body Radiation Therapy,^6^ AAPM Policy AP125‐A, QMP Supervision Levels,^8^ AAPM MPPG 10.a: Scope of Practice^9^ and ACR‐AAPM Technical Standard for Medical Physics Performance Monitoring for Stereotactic Body Radiation Therapy^10^ each contribute to the definition of a QMPs responsibility for many of the technical aspects of radiation treatment, including radiosurgery. These responsibilities include, but are not limited to, acceptance and commissioning of radiation oncology systems, implementing and managing QA programs, supervision of the treatment planning process and staff training.

The QMP's scope of practice includes administrative duties, clinical services, education, informatics, equipment performance evaluation, quality, and safety. Safety includes continued evaluation of the radiation safety infrastructure as the practice evolves. The scope of practice for an SRS‐SBRT program will include but is not limited to:

- Planning and acquisition of equipment/devices for the SRS/SBRT program.
- Performing acceptance testing and commissioning of the SRS‐SBRT system, including validation of the treatment planning system accuracy with small fields and tissue heterogeneities (if relevant to the scope of SRS‐SBRT services offered), accuracy of targeting through end‐to‐end (E2E) testing, quality and precision of the image‐guidance system and any motion management system, and validation of an associated record and verify system.
- Implementation and management of a QA program that ensures continuous proper performance of the treatment delivery unit, immobilization and simulation devices, image guidance system, treatment planning system, and patient‐specific QA software and devices. Work with other team members to develop standard operating procedures (SOPs) for major steps through the entire treatment process.
- Establishment of a comprehensive safety checklist to act as a guide for the entire treatment process, and determine appropriate methods for the clinic's QA committee to monitor the SRS‐SBRT program.
- Facilitation and management of the clinic's participation in an incident learning system to ensure a transparent, structured evaluation of all “near miss” and actual deviations in the planning and treatment delivery process.
- Performance or supervision of the dosimetric treatment planning process, providing supervision levels as appropriate to each task (e.g., direct supervision at the initial and final phases of the treatment planning process).
- Review the final treatment plan for accuracy and deliverability, consulting with the radiation oncologist to ensure that both professionals are confident of the acceptability of the chosen treatment plan.
- Validation of the chosen treatment delivery parameters via an independent dose calculation. When deemed appropriate, a phantom measurement or treatment delivery “dry run” may also be performed.
- Supervision of SRS‐SBRT treatment sessions by a QMP with relevant SRS‐SBRT training. For the first treatment session the QMP must provide personal supervision of the session.^2^ For any subsequent treatment sessions, direct supervision should be provided by either a QMP or a medical physicist under the supervision of a QMP. The level of physicist supervision must be considered under the guidance of the QMP based on the complexity of the procedure and the experience of the team with the particular technologies utilized.
- Establishment of a communication process to inform the QMP in the event that a patient experiences an unexpected patient side effect so, if necessary, the QMP can investigate potential causes.

### 3.3 Radiation oncologist

#### 3.3.1 Qualifications

The radiation oncologist should be certified in Radiation Oncology or Therapeutic Radiology by the American Board of Radiology and have completed specific training in SRS‐SBRT as stated in the ACR‐ASTRO Practice Parameter for Radiation Oncology,^11^ ACR‐ARS Practice Parameter for the Performance of Brain Stereotactic Radiosurgery^5^ and the ACR‐ASTRO Practice Parameter for the Performance of Stereotactic Body Radiation Therapy^6^ prior to commencing SRS‐SBRT services.

#### 3.3.2 Responsibilities

As stated in the ACR‐ARS Practice Parameter for the Performance of Brain Stereotactic Radiosurgery^5^ and the ACR‐ASTRO Practice Parameter for the Performance of Stereotactic Body Radiation Therapy.^6^

### 3.4 Medical dosimetrist

#### 3.4.1 Qualifications

A dosimetrist providing SRS‐SBRT treatment planning services should be certified by the Medical Dosimetry Certification Board. The dosimetrist must have specific training in SRS‐SBRT planning prior to performing patient‐specific procedures, and must be supervised by a radiation oncologist and QMP. The QMP, with responsibility for the SRS‐SBRT, program is responsible for determining the competency of each dosimetrist to provide SRS‐SBRT treatment planning services. *Note*: A medical physicist with appropriate training in SRS‐SBRT planning may perform the activities listed under section 3.4.2 below.

#### 3.4.2 Responsibilities

A dosimetrist should participate in simulation sessions as needed under the radiation oncologist's and QMP's supervision, delineate normal tissue volume, develop dosimetric treatment plans in accordance with the radiation oncologist's clinical objectives, document the chosen treatment technique, and ensure that all aspects of the chosen treatment technique are clearly conveyed to the therapist team. For unusual or complex aspects of a patient's treatment technique, the dosimetrist will communicate directly with the therapists to ensure that the therapist team is aware of these complexities and how it may affect patient setup and treatment.

### 3.5 Radiation therapist

#### 3.5.1 Qualifications

All radiation therapists should hold an active certification in radiation therapy by the American Registry of Radiologic Technologists (AART), should fulfill any state licensing requirements, and must have specific training in the clinic's SRS‐SBRT procedures prior to performing patient‐specific SRS‐SBRT procedures.

#### 3.5.2 Responsibilities

For each simulation session, prepare immobilization devices, position the patient, and acquire images for treatment planning in accordance with the clinic's SRS‐SBRT procedure and the patient‐specific instructions, and document treatment setup parameters after the radiation oncologist has approved the positioning, images, and target localization.

For each treatment session, prepare the treatment room for the SRS‐SBRT procedure in accordance with the clinic's SRS‐SBRT procedure and the patient‐specific instructions, position the patient and localize the treatment isocenter, and operate the treatment unit after the radiation oncologist and QMP have approved the clinical and technical aspects of the treatment delivery.

If the QMP has delegated certain daily QC tasks, perform the relevant QC tests under the QMP's direct supervision following the procedure established by the QMP, notifying the QMP of any performance deviations, warnings, failures, or other issues that may be detected.

## 4 RESOURCES

Because of the high dose per fraction and the critical importance of targeting accuracy, SRS‐SBRT services require a strong commitment by the radiation oncology program and the facility to provide the appropriate resources. Both the clinical team and the institution's administration must understand their roles and commitments prior to implementing SRS‐SBRT services. In this context, “resources” refers to appropriate staffing and coverage, appropriate equipment to support the treatment process, appropriate instruments for QC, clear operating procedures with delineation of duties and appropriate time intervals for all staff to safely perform their work, and a safety culture rooted in transparency and process analysis.^12^, ^13^, ^14^ Accrediting bodies such as the American College of Radiation Oncology (ACRO) and the American College of Radiology (ACR) provide resource data from their accredited facilities for reference.^15^, ^16^

An institution must not offer SRS‐SBRT services unless it can provide the following resources and support the following programmatic imperatives.

### 4.1 Staffing and coverage

1. Adequate medical physicist staffing to ensure that a QMP with appropriate SRS‐SBRT training is available to review QC results and consult with the clinical team on patient‐specific aspects of treatment planning and delivery and to provide personal supervision for the first treatment session of every SRS‐SBRT treatment course and as necessary for portions of all subsequent treatment fractions.
2. Adequate radiation oncologist staffing to ensure that a radiation oncologist with appropriate SRS‐SBRT training is available for direct supervision of the simulation, treatment planning, and treatment delivery of every SRS‐SBRT treatment course.
3. Adequate staffing to ensure that a dosimetrist or medical physicist with appropriate SRS‐SBRT training can devote the time necessary to develop a treatment plan with a comprehensive review of all technical aspects such as prior treatment, respiratory motion of target and adjacent organs, multi‐modality image registration, and potential limitations in treatment delivery.
4. Adequate therapist staffing to ensure that at least two certified radiation therapists are present for every treatment session, with at least one therapist who is appropriately trained in the SRS‐SBRT treatment technique being used.

### 4.2 Instrumentation

1. Redundant radiation detectors suitable for small fields.
2. Reference‐grade electrometer is suitable for low‐signal readings.
3. Appropriate End‐to End (E2E) phantoms for the scope of SRS‐SBRT services offered, available for use on site in a timely manner.
4. QC resource to perform a radiation isocentricity beam alignment verification.
5. QC device to verify off‐axis delivery accuracy for SIMT treatments.^17^, ^18^, ^19^

### 4.3 Simulation, planning, and treatment resources

1. Appropriate devices for patient setup and immobilization.
2. Appropriate devices for proper motion management.
3. Computerized treatment verification system.
4. Digital access to magnetic resonance imaging (MRI) and positron emission tomography (PET) image data.
5. Four‐dimensional computed tomography (4D CT) capability (for thoracic and abdominal SBRT services).
6. Multi‐modality image fusion capability.^20^
7. Capability to calculate, display, and evaluate composite dose for patients who have received prior radiation therapy.^21^
8. Linac‐based treatment delivery system with appropriate mechanical accuracy, field‐aperture size and resolution for small‐target conformality, and image‐guidance devices for target localization and verification including motion management technology relevant to the scope of SRS‐SBRT services to be offered.

### 4.4 Administrative support

1. Commitment to support the delineation of duties, procedure‐specific QA, and staff authority required for safe delivery of SRS‐SBRT services, as defined in standard operating procedures (SOPs) developed by the institution's QMP and medical director of radiation oncology consistent with the institution's credentialing process.
2. Commitment to facilitate and pay for independent peer review of the SRS‐SBRT program and on‐site proctoring of the first SRS‐SBRT treatment(s) if the clinical team does not have relevant prior experience with the SRS‐SBRT service being implemented at the center.^21^, ^22^, ^23^
3. Commitment to support ongoing training for members of the SRS‐SBRT team as deemed necessary by the medical director and QMP.
4. Robust preventive maintenance and field service support arrangements for the key simulation, planning and treatment delivery systems.

## 5 ACCEPTANCE TESTING AND COMMISSIONING

### 5.1 Acceptance testing

The QMP must be involved with the process of facility design, equipment selection, and specifications and provide direct supervision during the acceptance testing process.^7^ Customer acceptance test procedures are intended to ensure that the equipment satisfies the performance requirements stated in the purchase agreement and that the equipment is safe to operate. In some cases, measurements completed as part of the acceptance procedures may also serve as components in establishing the routine QA program. The vendor must demonstrate acceptable system performance.

### 5.2 Commissioning

To determine the scope of SRS‐SBRT commissioning, the QMP must understand the scope of procedures/services to be offered. Commissioning encompasses the overall process of validating the planning and delivery system for the services to be offered and developing appropriate QC and technical procedures to support these services. The scope of commissioning must, therefore, be commensurate with the scope of clinical services to be offered.

#### 5.2.1 Equipment commissioning

Each SRS‐SBRT system is highly specialized with fixed cones and/or multileaf collimators (MLCs) and ancillary systems for special techniques to achieve treatment delivery with high accuracy and precision. Specific validation must be based on manufacturer recommendations and the determined scope of the practice. Commissioning of such systems includes, but is not limited to, a safety and geometric accuracy evaluation of the treatment and imaging components, comprehensive small field data measurement with appropriate small field detectors and careful equipment setup, evaluation of treatment planning system capabilities including multi‐modality image processing and calculation accuracy for small fields, and the development of a comprehensive QA program for each of the following critical components:

- Treatment delivery machine.
- Immobilization equipment.^24^
- Ancillary systems for imaging^25^ and motion management.
- Treatment planning systems.^26^

##### Treatment delivery machine

Commissioning of a linac‐based treatment delivery system is performed after acceptance testing. Commissioning tests must be developed by the institution's medical physics team to explore in detail every aspect of the system with the goal of developing a comprehensive characterization of the performance of the system, identifying any limitations relative to clinical use, and developing procedures for QA and clinical use.^7^, ^27^, ^28^ A variety of task group reports are referenced in this document to provide guidance on best practices for performing commissioning and QA of delivery devices. However, SRS‐SBRT intent requires special consideration.

Gantry, collimator, and couch isocentricity must be verified during the acceptance and commissioning to meet the required specifications for SRS‐SBRT treatment. The vendor must demonstrate the isocentricity using multiple gantry, collimator, and couch angles (including pitch and roll for a 6‐degree freedom function) and prove the confined isocenter radius is within the criteria. Also, the QMP must confirm the results using an independent isocentricity test including off‐axis verification, for example, Gafchromic film or EPID‐based Winston‐Lutz (WL) test.^17^, ^18^, ^19^

Small field dosimetry as used in SRS‐SBRT is challenging due to many factors, including source size, detector size, and detector response.^29^ As a generalization, even micro‐ion‐chambers are large relative to the field sizes used in SRS‐SBRT due to a lack of charged particle equilibrium.^30^, ^31^ Generalized approaches to the lack of lateral equilibrium, and violation of cavity theory have been addressed in the literature.^32^, ^33^, ^34^, ^35^ Newer solid‐state micro‐detectors have become available such as a diodes, plastic scintillators, and synthetic microdiamonds that have shown appropriate characteristics for small field dosimetry. Evaluations of many commercially available detectors have been published with correction factors for small‐field dosimetry.^36^, ^37^, ^38^

A practical measurement methodology for validating small‐field beam data using multiple detectors has also been reported.^39^ A published code of practice from the International Atomic Energy Agency TRS‐483^38^ in conjunction with TG155^37^ provides a useful guideline for small‐field dosimetry. An important characteristic of any detector used for commissioning is that the detector's active area is of small size compared to the field size range to be characterized. A daisy‐chain method is recommended, using two independent detectors with one of them suitable for measuring small fields. The included references contain detailed information regarding detector selection and measurement methodology. Understanding and implementing the appropriate detector and methodology is critical.

Upon completion of beam data measurements, key data points (such as percent depth dose at 10 cm depth and output factors for field sizes ≤2.0 cm) must be compared to other machines of identical design, whether in the same institution or from other centers, to guard against gross errors which could arise from inappropriate detector selection or misaligned equipment setup.

##### Immobilization equipment

The smaller margins utilized in SRS‐SBRT treatment planning necessitate immobilization that is highly accurate and reproducible. The equipment must be evaluated for its effectiveness in targeting accuracy and precision (e.g., through analysis of shifts after pretreatment imaging for a representative sample of patients for each treatment site), and must be evaluated for its beam attenuation and surface dose characteristics.^24^, ^40^, ^41^ The effects of attenuation, surface dose, and compatibility with surface guided radiation therapy systems (SGRT) must be clearly articulated to the clinical team prior to the implementation of the clinical service.

##### Treatment planning system

Commissioning of the treatment planning system's dose calculation model must include all aspects described in the latest revision of AAPM Medical Physics Practice Guideline 5.^26^ For SRS‐SBRT, clinically relevant small field dose calculations (using cone, Iris or MLC fields if in scope) must be validated by comparing doses computed in the TPS clinical module to measurements obtained during TPS commissioning.^42^, ^43^ Additional validation tests must be performed as appropriate for the specific SRS‐SBRT delivery technology and scope of clinical services such as evaluation of multi‐modality image fusion accuracy, high‐resolution segmentation accuracy, calculation accuracy for attenuation and effect on surface dose from couch and immobilization devices,^24^ and heterogeneity corrections. Dose calculation algorithms must be appropriate for extracranial SRS‐SBRT applications where high accuracy and precision are required and the beam paths traverse significant tissue heterogeneities, such as for lung, dome of liver, and nasopharynx treatment sites.^44^, ^45^ In 2020, RO‐ILS published a Safety Notice together with a more in‐depth study regarding the commissioning of heterogeneous dose calculation for SRS.^46^

##### Ancillary systems for imaging and motion management

CT simulators must be commissioned according to the recommendations of the AAPM Task Group 66 report^25^ with the understanding that additional testing and stricter action limits may be appropriate for simulators used in SRS‐SBRT applications. The geometric fidelity, spatial resolution, and slice thickness requirements for SRS‐SBRT applications may be more strict than those of conventional treatments.^4^ X‐ray image guidance systems (X‐IGRT) must also be assessed for suitability in SRS‐SBRT. AAPM MPPG 2.b states that the composite tolerance of the imaging‐treatment isocenter coincidence and table repositioning accuracy must be ≤1 mm for SRS and ≤1.5 mm for SBRT, tested daily when SRS‐SBRT treatments are conducted.^47^ Imaging systems commissioned prior to the inception of an SRS‐SBRT program must be reevaluated.

MR‐simulators have been increasingly used for SRS‐SBRT applications. Geometric distortions inherent to MR images are a concern, and it is the responsibility of the QMP to ensure that these have been characterized. Guidance for the commissioning and use of these systems is provided in the AAPM Task Group 284 report.^48^

SGRT systems can be used for patient setup, intrafraction motion monitoring, and respiratory gating. For frameless intracranial SRS cases, open‐face thermoplastic masks allow for the patient's face to be used as a reference surface. The AAPM TG302 report provides recommendations for the commissioning, QA, and clinical implementation of SGRT systems, including specific recommendations for SRS‐SBRT applications.^49^ QA recommendations for SGRT and other non‐radiographic localization systems can be found in Table 5 of this report.

If the planned scope of clinical services includes treatments affected by respiratory motion, the entire treatment chain (CT simulation, treatment planning, treatment delivery) should be assessed with E2E testing using a dynamic phantom setup with clinically relevant motion parameters (amplitude, cycle time). The tests should include an assessment of spatial targeting accuracy and measurement of the delivered target dose.^50^ Dynamic phantoms are available for commissioning this service if one is not available on site.^44^

##### End‐to‐end test

The E2E tests must be designed to check the performance, accuracy, and connectivity of all equipment within the SRS‐SBRT workflow, including simulation, respiratory management, treatment planning, and treatment delivery using a clinically relevant image‐guidance method. In addition to E2E testing, the beam model in the treatment planning system and machine output calibration must be validated independently prior to the initiation of the SRS‐SBRT programs. The beam model can be validated using an independent dose calculation check. Both the beam model and machine output can be checked through E2E phantom tests from the Imaging and Radiation Oncology Core (IROC),^44^ or through an independent physicist's on‐site review.^23^

##### Commissioning report

The scope of the commissioning work and key results must be summarized in a written commissioning report. The report must clearly identify known limitations in the delivery chain, limits for clinical implementation (e.g., minimum field size), and define the primary reference data to support the equipment QC program. If the full commissioning report is not completed prior to initiation of the clinical service, an Executive Summary describing the known limitations and limits for clinical implementation must be prepared and shared with the clinical team prior to initiation of the clinical service.

#### 5.2.2 Process commissioning and clinical implementation

Clinical implementation of a stereotactic program requires agreement within the clinical team on the scope and clinical goals of the program. Development and validation of the technical process to be followed for delivering a clinical SRS‐SBRT service may be regarded as process commissioning and must be completed prior to the first patient treatment. The SOPs for each anatomical site to be treated should be developed in collaboration with the clinical team. That team includes the radiation oncologist, the QMP, the medical dosimetrist, the radiation therapist and often the radiation oncology nurse. There are several references available, including AAPM task group reports^27^, ^51^ ACR‐ASTRO Practice Parameters,^5^, ^6^ ASTRO^52^ and recent AAPM Medical Physics Practice Guidelines.^26^, ^47^, ^53^ Each of these references should be reviewed to develop an overall understanding of the scope of stereotactic program implementation.

Specific clinical implementation guidance is found in Section VII of the AAPM TG101 report.^27^ These components described in the TG101 report are also applicable to SRS procedures. The following section is consistent with the TG101 recommendations, providing additional details deemed relevant to a clinical SRS‐SBRT program.

##### Standard operating procedures (SOP)

SRS‐SBRT SOPs must address the components essential to the patient review, simulation, planning, treatment, and follow‐up (*see the* *Appendix B* *for a detailed sample SOP document*). A general SRS‐SBRT SOP can be supplemented with site‐specific details when necessary. QMPs must determine the detail needed to document the practice. Patient safety must be the primary consideration when developing any SOP.

(1) Safety
  - The roles and responsibilities of each member of the clinical team must be clearly described in the SOP document. See section 35 of this Practice Guideline for additional information.
  - Mechanical operational limits and action limits will be established during commissioning and must be well documented. Additional tolerances for clinical operation should be considered for each SRS‐SBRT service and should be clearly defined in the SOP document.
  - The SOP must establish certain process expectations for safe implementation, such as appropriate time intervals from simulation to treatment with critical points along the path allowing for reconsideration or rescheduling.
  - The SOP must clearly state that every team member has the right and responsibility to halt a case and/or a particular procedure based on safety imperatives.
(2) Patient selection
  - Patient selection criteria should initially be determined using data available from clinical protocols or published guidelines. Minimum and maximum target sizes should be documented along with standard prescription dose and fractionation schemes.
  - Where possible, a multidisciplinary review or a peer review of proposed cases should be completed prior to simulation. If the patient is enrolled in a clinical trial, the rules and guidelines of the clinical trial must be followed.
(3) Simulation
  - Reproducible immobilization techniques must be developed for each treatment site.
  - The reference imaging study to be used for treatment planning must cover the target and all relevant organs at risk and any relevant immobilization device that may be utilized by the treatment planning system to account for attenuation. A typical scan length should extend at least 10 cm beyond the treatment field borders. For non‐coplanar treatment techniques, the scan length may need to be further extended to adequately model the beam paths and resultant scatter dose^27^ and extend beyond the entrance path and clinically relevant exit path of every beam.
  - For SBRT applications, a tomographic slice thickness of 1–3 mm must be used. For SRS applications, slice thickness should not exceed 1.25 mm, and the scan field of view should be optimized for maximum in‐plane spatial resolution while including all necessary anatomy and immobilization hardware in the field of view.
  - Respiratory motion management must be considered in thoracic and abdominal sites. At least one of the five categories of motion management, as described in the AAPM Task Group 76 report^50^ should be implemented, with a QA program consistent with the TG76 recommendations. The TG76 report includes a flowchart for assessing and managing respiratory motion.
(4) Treatment planning
  - The treatment planning system must have the capability of accurately calculating the predicted dose for the scope of SRS‐SBRT services to be offered.^26^, ^27^
  - Each treatment site should have a defined list of critical structures using standardized nomenclature.^54^ Stereotactic fractionation‐based tolerances should be defined based on clinical protocol data or peer reviewed literature.^55^ The QMP must ensure that the radiation oncologists are aware of the delivery system's tolerances relative to the planning target volume and organs at risk avoidance margins. The planning target volume margins must be clearly documented.
  - Image fusion requirements for target definition must be defined and target margins clearly described. Clear guidelines provided to the personnel performing image registration and registration accuracy should be assessed regularly based on the policy.^20^ Target margins should be based on data from current literature along with knowledge of the limitations of in‐house localization capabilities.
  - Planning strategies and techniques should be described for each treatment site, such as conformal arcs, intensity‐modulated radiation therapy, and volumetric‐modulated arc therapy. These technique definitions must include clinical limitations based on the findings from commissioning. If non‐coplanar techniques are included, the potential collision must be considered in determining the overall beam configuration.
  - In cases of re‐irradiation, the prior radiation target structures and plan should be made available to the radiation oncologist and physicist. If plans are not available or obtainable expeditiously, attempts must be made to acquire other documentation of the previous treatments, such as physician notes and plan documents. Tailored dose constraints which make use of EQD2 dose summation are appropriate for re‐irradiation cases. Tailored dose constraints may then be used in plan optimization, or de novo constraints may be used at the discretion of the radiation oncologist. A report should be generated including the cumulative dose evaluated, a description of the method used for the evaluation as well as the results.^56^
  - In the case of multiple brain metastases and re‐irradiation, a target naming convention that makes clear which targets are new and which are old must be utilized; for example, include the date of the MR scan at the end of the target structure's name.
  - The use of an isotropic calculation grid size of 2 mm or finer should be utilized. The use of a grid size ≥3 mm is discouraged.^27^ For very small targets (≤1 cc) or targets with adjacent critical organs at risk (OARs), a 1 mm calculation grid size may be necessary.
  - Target dose coverage, dose fall‐off beyond the target, dose conformity metrics, and compliance with critical structure dose objectives^55^ should be clearly reported and signed by the radiation oncologist to confirm that the chosen treatment technique is clinically acceptable.
  - An independent dose calculation check must be performed prior to treatment.^57^, ^58^
  - An initial plan/chart review by the QMP must be performed before the treatment based on the TG275 and MPPG 11.a recommendation to enhance patient safety and quality of care.^59^, ^60^ The check prior to treatment is critical in SRS‐SBRT due to the high dose and number of fractions utilized.
(5) Treatment delivery
  - A clearly defined pre‐treatment QA check must be performed and may depend on the technique used^61^ (e.g., frameless cranial, frame‐based cranial, cone‐based SRS, or SBRT). This should include a “dry run” collision check where the potential for collision exists.
  - The SOP should clearly describe the professional supervision requirements for each SRS‐SBRT treatment type.^5^, ^6^, ^8^, ^27^, ^51^
  - The SOP should clearly describe the image‐guidance method to be used, including target anatomy, critical organ avoidance, and localization tolerance. Pre‐treatment verification of target localization must always be performed; the criteria for intra‐treatment image guidance should be clearly described.^62^
  - If motion management is used, the SOP should clearly describe the process, tolerances, and professional supervision.
(6) Checklists
  - Effective checklists support human thinking, allow constructive team member interactions, and facilitate a systematic care delivery by reducing process variability. MPPG 4.b^53^ and MPPG 11.a^60^ should be followed in developing treatment‐specific checklists.

##### Training

The program must address the need for initial as well as ongoing training and must be supported by a system of documentation and checklists to ensure that all team members are competent to support the clinical service^.^ ^22^

(1) Training venues
  - Vendor training—A core team should participate in all available vendor training on relevant hardware and software including off‐site training, on‐site training, and case observation.
  - Non‐vendor training—Attendance at structured courses and/or “shadowing” procedures at a facility with a mature SRS‐SBRT service should be considered. This should include a review of the SOP for the SRS‐SBRT services to be implemented, including equipment specific and patient‐specific QA procedures.
  - If the principal professionals responsible for the SRS‐SBRT service do not have direct prior experience with the services to be offered, the facility must arrange for on‐site review and proctoring of the first clinical procedure by professionals with experience relevant to the new service.^23^
(2) Continuing education
  - Continuing Education must target educational opportunities related to SRS‐SBRT.^2^
  - Routine review of incident learning should be used to leverage learning from past incidents.^63^, ^64^
  - Plan review and chart rounds (peer review) should occur prior to treatment and during the first week of treatment, respectively, serving as a QC measure involving multidisciplinary peer review. Due to the short fractionation of SRS‐SBRT, effort should be made to review plans prior to treatment.^65^
  - Quality review forum or in‐service can be utilized to promote quality and patient safety within the care team
(3) Documentation
  - Written SOPs must be developed and reviewed by all participating staff.
  - All training must be documented.
  - A checklist of relevant competencies should be developed and completed prior to program implementation. A competency checklist must be reviewed periodically and updated as the program evolves.^22^

##### Process validation

To assess the clinical team's readiness and to validate the SOP, the team should develop a process map to visualize the entire process and cross‐check each category of SRS‐SBRT service in the SOP against the process map (*see* *Appendix C* *for an example of a process map*). The E2E testing mentioned in the equipment commissioning section should also be performed following the flow of the process. Developing safety checklists during this process validation phase is recommended. Implementing safety checklists for each category of SRS‐SBRT service will ensure the team adheres to critical steps and provides consistent performance.^53^

## 6 QUALITY ASSURANCE

### 6.1 Introduction

A comprehensive QA program for SRS‐SBRT is critical to ensure the correct dose is delivered to the target, given the very small target volumes and rapid dose fall‐off associated with SRS‐SBRT. QA processes and procedures related to SRS‐SBRT must be designed to cover the following aspects of the SRS‐SBRT program: equipment‐specific QA, patient‐specific QA, and procedure‐specific QA. Safety and QA recommendations have been extensively described in several publications.^5^, ^6^, ^27^, ^61^

When equipment performance is found to exceed action limits, the affected module(s) of the delivery system must be promptly adjusted, and the QMP must verify proper performance before clinical SRS‐SBRT services resume. In the event of a significant service interruption, the QMP must coordinate closely with treating physicians to evaluate the impact on patients’ treatment schedules given the importance of completing SRS‐SBRT treatment courses in a short overall time interval (generally 14 days or less).^66^ Patient safety must be the primary consideration in determining when to resume clinical services.

Tolerance limits define the bounds of normal operating range including an allowed expected variation. Action limits are thresholds that, if exceeded, may significantly impact patient care. This document continues the use of these definitions as described in MPPG 8.b. Exceeding tolerance limits requires timely investigation and intervention. Exceeding action limits requires immediate intervention.^7^

### 6.2 Equipment‐specific QA

#### 6.2.1 Treatment machines

The AAPM has published task group reports with recommendations for QA related to SRS‐SBRT. TG142, TG198, and MPPG 8.b describe the linear accelerator QA and together provide testing methods for both conventional radiation therapy QA procedures and for SRS‐SBRT QA procedures.^7^, ^67^, ^68^ MPPG 2.b^47^ provides recommendations for commissioning and QA of X‐ray‐based image‐guided radiotherapy systems. TG135 provides specific guidance for QA of robotic radiosurgery systems.^51^ TG148 and TG306 provide specific guidance for QA of helical tomotherapy systems.^69^, ^70^ MPPG 5.b provides minimum QA recommendations for treatment planning system dose algorithms.^26^ TG50b and TG332 are in progress and will be additional QA resources. As mentioned in Section 5, the device baselines for routine equipment QA (daily, monthly, and annual QA) must be established during machine commissioning and initial calibration.

The SRS‐SBRT relevant QA tests, frequencies, and action limits are summarized in Tables 1, 2, 3 below, for C‐arm linac, robotic linac, and ring‐mounted helical tomotherapy systems, respectively. All three tables are updated based on the recent publications. QA recommendations on the recently introduced Halcyon and Ethos systems are significantly different from the previous C‐arm and helical systems,^7^, ^67^, ^71^, ^72^, ^73^ therefore, Table 4 summarizes the QA recommendations separately for Halcyon and Ethos.

**TABLE 1:** Minimum SRS‐SBRT relevant equipment QA and action limits for C‐arm linac systems.

Frequency	Test	Action limit
Daily	Accelerator output constancy^7^	±3% of device baseline
	Beam profile constancy^7^	±2% of device baseline
	Laser localization—**only** if using SRS techniques relying on lasers for target localization (e.g., frame‐based SRS without X‐ray IGRT)	1 mm
	Radiation isocentricity test (limited gantry and couch positions)—maximum deviation in center of target object relative to each projection's beam central axis	1.0 mm SRS, 1.5 mm SBRT
	MLC position accuracy (at least weekly but it is recommended to develop a daily simplified qualitative test or utilize available automation)	0.5 mm
	IGRT/SGRT positioning/repositioning	1 mm SRS, 2 mm SBRT
	Imaging subsystem interlocks	Functional
	Stereotactic interlocks—cone size, backup jaws	Functional
Monthly	Photon output constancy^7^	±2% of device baseline
	Photon dynamic delivery control^7^	±2% of device baseline
	Radiation isocentricity test—covering complete range of gantry, couch, and collimator rotations used clinically—calculated 3D displacement. *Note: If both MLC and fixed conical collimators are used, both must be evaluated at least monthly or prior to use	1.0 mm SRS, 1.5 mm SBRT max 3D displacement from center of target object.
	Treatment couch position indicators: relative over the maximum clinical range for SRS/SBRT	1 mm/0.5 degrees
Annually	Photon output^7^	±1.0% of nominal TG51 output
	Photon profile constancy^7^ (spot check with TPS model‐based profiles)	±2% of TPS OAFs
	Photon output vs. dose rate^7^	±2% of output for TG51 dose rate
	MU linearity (≥5 MU to highest MU used clinically)^7^	±2% of nominal
	Verification of relative output factors for cones and/or small field MLC defined fields in the range of clinical use for SRS‐SBRT.	±2% from TPS for > 1.0 cm apertures, ± 5% from TPS for ≤1.0 cm apertures
	Coincidence of radiation and mechanical isocenter Note: If both MLC and fixed conical collimators are used, both must be evaluated.	±1.0 mm maximum 3D displacement from center of target object.
	MLC transmission	5% from TPS
	SRS arc rotation mode (if used clinically)	1 MU, 1 degree
	E2E localization assessment “hidden target test” using SRS frame and/or IGRT system	1.0 mm
	E2E dosimetric evaluation using SRS frame and/or IGRT system. Absolute dose measurement to a point and fluence evaluation.	±5%, gamma (3%, 1 mm) > 90%

*Note*: Action limits are absolute accuracy, not variation from baseline, unless otherwise stated.

**TABLE 2:** Minimum equipment QA and action limits for robotic linac systems.

Frequency	Test	Action Limit
Daily* *On days of clinical use	Accelerator output constancy	±3%
	Head laser alignment check	1.0 mm
	Picket fence for MLC	visual check
	Automatic QA (AQA) test* *If the clinic has more than one fixed cone, Iris and MLC collimator, the AQA test should alternate through fixed cones, Iris, and MLC with each system tested at least weekly	Total targeting ≤1.0 mm from baseline
	Safety interlocks	Functional
Monthly	Accelerator output constancy	±2%
	Energy constancy	±2%
	Beam symmetry, relative	±2% largest field size
	Garden fence for MLC (*if applicable*)	>90% deviations < 0.5 mm, mean deviation < 0.2 mm, all < 0.95 mm
	Iris field size spot check	0.5 mm, 3 or more field sizes ≥10 mm
	Imager alignment	1 mm or center pixels ± 2 pixels
	E2E localization assessment (cover each collimator monthly, rotate through tracking modes, Test Synchrony quarterly)	1.0 mm static target, 1.5 mm motion tracking
Annually	Accelerator output	±1.0%
	MU linearity (>10 MU to highest MU used clinically)	±2%
	Path verification	≤0.5 mm maximum per node, ≤0.3 mm average, minimally, not exceeding manufacturer's specification
	Beam laser and radiation beam alignment for cone, Iris and MLC	0.5 mm from baseline, not exceeding manufacturer's specification
	MLC transmission	Max transmission < 0.5%
	AQA baseline	Re‐check AQA baseline
	Beam data verification – Relative output factors for cones, Iris and/or MLC covering the range used clinically	±2% from TPS for > 1.0 cm apertures, ± 5% from TPS for ≤1.0 cm apertures
	TPR or PDD constancy Profile constancy Note: Beam data checks on at least three field sizes per collimator assembly, including largest and smallest clinically relevant field sizes, and compared to commissioning data.	±1% from baseline (commissioning data) for apertures > 2.0 cm, ± 2% for apertures ≤2.0 cm ± 2% from baseline (commissioning data)
	Imager kVp accuracy, mA station exposure linearity, isopost alignment with center pixel	±10%, ± 20%, and 1 mm respectively
	Emergency power off (EPO) button, safety interlocks	Functional
	End to end dose verification	±5%, gamma (3%, 1 mm) > 90%

*Note*: Terminology and limits are intended to be consistent with TG135 (update in progress) and Accuray Physics Essential Guide with some adaptation for consideration of terminology in MPPG 8.b. Action limits are absolute accuracy, not variation from baseline, unless otherwise stated.

**TABLE 3:** Minimum SBRT relevant equipment QA and Action limits for helical tomotherapy systems.

Frequency	Test	Action Limit
Daily	Accelerator output constancy (rotational or static)	±3%
	Red laser initialization (congruence with green laser)	1 mm
	Image/laser coordinate coincidence	1 mm
	Image registration/alignment	1 mm
	Checking target offset detection accuracy (on the day Synchrony is used)	1 mm
	Checking the function of the jaw and MLC tracking (on the day Synchrony is used)	Point dose within 3% of baseline
Monthly	Accelerator output constancy and rotational output variation	±2%
	Beam quality constancy	±1% PDD_10_ or TMR^20^_10_
	Transverse beam profile	1% average difference in field core
	Longitudinal beam profile (each slice width)	1% of slice width (Symmetric jaw), 0.5 mm (asymmetric jaw)
	Red and green laser alignment	1 mm
	Couch positioning accuracy	1 mm
	Leaf Projection time error consistency with TPS MLC model (latency offset)	Within 2.31 ms of latency
Annually	Accelerator output	±1.0%
	Beam quality (5 cm slice width)	±1% PDD_10_ or TMR^20^_10_
	Couch speed uniformity	±2% dose nonuniformity
	Couch translation per gantry rotation	1 mm per 5 cm
	CT dimensional accuracy	1 mm
	Checking the Synchrony imaging and treatment using jaw and MLC tracking coordinate coincidence	2 mm (non‐SRS)
	E2E localization assessment “hidden target test”	1.0 mm
	E2E dosimetric evaluation	±5%, gamma (3%, 1 mm) > 90%

*Note*: Action limits are absolute accuracy, not variation from baseline, unless otherwise stated.

**TABLE 4:** Minimum SBRT relevant equipment QA for Varian Halcyon and Ethos systems.^7^, ^67^, ^71^, ^72^, ^73^ ^.^

Frequency	Test	Action limit
Daily	Accelerator Output Constancy	±3%
	Vendor required performance (MPC) test—used to evaluate MLC position and reproducibility, couch and gantry positioning, and virtual laser to isocenter shift (4)	Pass
	IGRT positioning/repositioning	2 mm
Weekly	MLC Picket Fence tests—distal MLC, proximal MLC and dual layers at all cardinal angles	1 mm
Monthly	Accelerator Output	±2%
	MLC based radiation isocentricity test for gantry and collimator angles used clinically	1.5 mm
	Dynamic Delivery Control Test	3% of open field dose
	Relative and absolute couch position	Abs: 2 mm Rel: 1 mm over 10 cm
Annually	Accelerator output	±1%
	Small field output factors	±2% from baseline for > 1 cm ± 5% from baseline for ≤1 cm
	MU linearity	±2%
	MLC leaf transmission—distal, proximal and both banks	±5% from baseline
	Lasers (virtual‐iso) versus machine treatment isocenter (independent from vendor provided)	±1 mm
	E2E hidden target test	1.0 mm
	E2E dosimetric evaluation using IGRT system	±5% measured versus calculated

*Note*: Action limits are absolute accuracy, not variation from baseline, unless otherwise stated.

#### 6.2.2 Patient setup and imaging systems

Surface guidance technologies have been adopted widely in SRS/SBRT applications in the past few years. QA recommendations are provided by TG302 and TG147.^49^, ^72^ A QA summary is added in Table 5.

**TABLE 5:** Surface Guided Radiotherapy (SGRT) *and other non‐radiographic localization* Systems Tests—Adapted for SRS/SBRT from TG302^48^ and TG147.^74^.

Frequency	Test	Action limit
Daily	Safety Interlocks	Functional
	Camera FOV clear check	Pass
	Static localization at isocenter (VRT Plate Verification)	2 mm (1 mm on VRT)
Monthly	Machine interfacing	Functional
	Hidden target test (Cube cal on VRT)	1 mm
	Dynamic localization of couch	2 mm
	Couch walkout with respect to system isocenter	1 mm
Annually	Test/reset buttons, backup power, emergency off switch	Pass
	Camera mounting integrity	Pass
	Camera settings	Same as commissioning values
	Drift measurement over at least 1 h	<2 mm over 1 h
	Reproducibility of localization	<1 mm after stabilizing
	Complete end‐to‐end test	<1 mm from isocenter
	Translation and rotation auto‐correct over a clinical range of motion	<2 mm from isocenter
	Radiation dosimetry accuracy using a motion phantom to test gating system functionality	<2%
	Data transfer to and from all systems	Functional

*Note*: Action limits are absolute accuracy, not variation from baseline, unless otherwise stated.

In addition, each CT scanner used for treatment planning should be evaluated at least annually to confirm geometric accuracy^25^ and constancy of the CT‐number to density curve.^26^

MR simulation and guided treatments are increasingly utilized as part of SBRT and SRS planning and treatment. TG284 provides guidelines for QA for MR imaging for simulation.^48^ AAPM Task Group 352 is working to establish formal guidance and tolerances for QA of MR‐guided linacs. ICRU Report 97 and existing vendor‐specific published guidelines provide further details for frequency, performance, and tolerances for QA of MR‐guided linacs.^75^, ^76^

With favorable outcomes from multiple studies of SRS in the treatment of limited brain metastases, treatment using SIMT has been increasing on C‐arm linacs in the past few years. However, it is crucial to acknowledge that angular uncertainties in patient positioning and delivery can potentially lead to additional dose errors in off‐centered targets.^75^, ^76^, ^77^, ^78^, ^79^, ^80^ Therefore, it is important to establish additional QA and clinical protocols prior to its clinical application. In addition to the QA covered in TG142 on couch, collimator, and gantry rotations,^67^ it is essential to implement an integrated QA process. Recent research^18^ underscores the sensitivity of the off‐axis WL test to angular errors, and commercially available QA phantoms designed for off‐axis WL testing have been developed. It is recommended that each institute develop their own off‐axis targeting assessment policy based on available publications^19^ until the recommendations of the TG362 Multi‐lesion Stereotactic Radiosurgery report are available.

***Note***: Many tests described in the aforementioned AAPM publications are important for characterizing the system performance regardless of the scope of clinical use; the equipment‐specific QA in Tables 1 through 5 are those deemed most directly relevant to the SRS‐SBRT service. The QMP responsible for the clinic's QA program should consider all recommendations in the aforementioned AAPM publications for their relevance to the clinic's overall scope of services.

### 6.3 Patient‐specific QA (PSQA)

#### 6.3.1 Overview

Compared with conventionally‐fractionated radiotherapy, the target volume in SRS and SBRT is much smaller, the dose heterogeneity is higher and the dose falls off faster in tissue. The term “Patient‐specific QA” for SRS and SBRT, in the context of this Practice Guideline, refers to verifying that the approved treatment plan can be accurately delivered.

#### 6.3.2 Scope of PSQA

Patient‐specific QA should include verification of patient setup/immobilization, independent check of the approved treatment plan and associated treatment delivery parameters, dose delivery measurements when appropriate, chart rounds and/or peer review, and a dry‐run of the approved treatment plan to check for potential collision. If fixed conical collimators are used, PSQA is prudent to verify the integrity of treatment, but is not essential since the measured dosimetric characteristics (profile, output, Tissue Phantom Ratio, etc.) are directly applied to the dose calculation. When the MLC is applied to modulate the dose, PSQA should be performed prior to treatment to verify the absolute dose to the reference point (usually isocenter) and an assessment of the dose distribution. Recommendations on tolerance limits and QA methods can be reviewed in the TG218 report.^81^ A tolerance limit with a 3% dose criterion relevant to the performance characteristics of the PSQA equipment and a 1 mm distance to agreement should be considered for SRS‐SBRT. An Institutional SOP based on the scope of services provided must be developed by the QMP for evaluation of plans that do not meet established PSQA criteria.

#### 6.3.3 Instrumentation for PSQA

The QMP determines the instrumentation appropriate to the SRS‐SBRT technique to be verified. Common instrumentation includes radiochromic film, small‐volume ion chamber (for relatively larger treatment fields), diode detector, portal imaging device calibrated for dose response, detector arrays and, less commonly, polymer gel dosimetry. The institution must provide appropriate PSQA instrumentation with consideration for adequate special resolution appropriate to the scope of the clinical service.^82^, ^83^, ^84^, ^85^ The clinical service should not be initiated if appropriate instrumentation is not available for the QMP's use.

### 6.4 Procedure‐specific QA

Procedure‐specific QA addresses issues related to operational tasks, such as checking whether:

1. The workflows to perform SRS‐SBRT as defined in the SOP documents are consistently followed.
2. Staffing level is appropriate.
3. Staff training and continuous training are available and appropriate.
4. Proper follow‐up actions are taken for any actual and/or potential (“near miss”) treatment incidents.

As described in the Clinical Implementation section above, each facility must have SOP documents defining the workflow of each SRS‐SBRT service. These documents must be reviewed and updated regularly, with at least an annual frequency of review.

Staffing levels, training, and competency assessments are critical for a successful SRS‐SBRT program. Team members without prior relevant SRS‐SBRT experience should perform a minimum of five procedures working under the supervision of an experienced expert for each SRS‐SBRT service.

Ongoing competency assessment is necessary given the rapid evolution of technology and treatment methods for SRS‐SBRT. These activities should be properly documented.

### 6.5 Quality management (QM) program supervision

The QM program should be designed by a QMP who has specific training in SRS‐SBRT, and should be reviewed by another QMP with SRS‐SBRT experience. The daily QC procedures can be performed by a physicist or radiation therapist and be reviewed by the QMP prior to any SRS‐SBRT treatment. Other routine QC or patient‐specific QA may be performed by an appropriately trained medical physicist, and reviewed and co‐signed by the QMP.

### 6.6 QM program review

When the SRS‐SBRT program is in its initial phase, the QM program should be reviewed semi‐annually as the clinical practice and utilization evolve. The frequency can be reduced to annual reviews once the clinical practice and utilization stabilizes.

## 7 CONCLUSIONS

While all team members are responsible for the delivery of SRS‐SBRT services, they work under the supervision of the radiation oncologist and QMP. Clinical procedures are supervised by the radiation oncologist and technical procedures are supervised by the QMP. The provision of SRS‐SBRT services should follow a structured approach with clearly defined roles, responsibilities, procedures, and action levels. The clinic's QMP develops SOPs for SRS‐SBRT through an active collaboration with the clinic's medical director, administrators, and clinical colleagues.

A failure mode and effects analysis (FMEA)^4^ should be completed for critical process pathways to provide the essential information needed to support the establishment of an SRS‐SBRT QA program.

The resources and programmatic components described in Section IV are imperative to the safe implementation of SRS‐SBRT services.

The scope of the commissioning work and key results must be summarized in a written commissioning report. The report must clearly identify known limitations in the delivery chain, limits for clinical implementation (e.g., minimum field size), and data to support the equipment QC program. Any relevant limitations must be clearly communicated to the clinical team prior to program implementation.

All new SRS‐SBRT programs must have independent validation of the beam model and machine calibration prior to initiation of the clinical service. If the principal professionals responsible for the SRS‐SBRT service do not have direct prior experience with the services to be offered, the facility must arrange for on‐site review and proctoring of the first clinical procedure by professionals with experience relevant to the new service.

## AUTHOR CONTRIBUTIONS

This guideline was reviewed and updated by the Medical Physics Practice Guideline Working Group on AAPM‐RSS MPPG 9.b: SRS‐SBRT of the Professional Council of the AAPM. Each author reviewed recent literature on the topic and offered opinions on and language for the guideline. They also reviewed and applied comments from the full AAPM membership to the document.

## CONFLICT OF INTEREST STATEMENT

The Chair of the Working Group on AAPM‐RSS MPPG 9.b: SRS‐SBRT has reviewed the required Conflict of Interest statement on file for each member of Working Group on AAPM‐RSS MPPG 9.b: SRS‐SBRT and determined that disclosure of potential Conflicts of Interest is an adequate management plan.

The members of Working Group on AAPM‐RSS MPPG 9.b: SRS‐SBRT listed below attest that they have no potential Conflicts of Interest related to the subject matter or materials presented in this document.

Eileen Cirino, MS, FAAPM, FACMP, Chair.

Stanley H. Benedict, PhD, FAAPM, FACMP.

Pamela J. Dupre, MS.

Meral L. Reyhan, PhD.

Christopher W. Schneider, PhD.

Lei Wang, PhD.

Carl P. Weaver, MS.

Sua Yoo, PhD.

Nicholai, Wingreen, BA.

The members of Working Group on AAPM‐RSS MPPG 9.b: SRS‐SBRT listed below disclose the following potential Conflicts of Interest related to the subject matter or materials presented in this document.

Per H. Halvorsen, MS, FAAPM, FACR is employed by Varian's professional services division to perform physics consultation related to implementation of advanced technology.

Grace Gwe‐Ya Kim, PhD, FAAPM is a Consultant for Varian Medical Systems.

## Supporting information



Supporting Information

## Footnotes

1 SRS using ring‐mounted delivery systems was excluded from the scope of this document due to the infrequent use of this technology for SRS. Exclusion from the scope of this document does not imply any AAPM position regarding the appropriateness of delivering such treatments using ring‐mounted delivery systems.

2 All treatments must occur under the supervision of a QMP. In addition, a QMP must provide personal supervision at the first treatment, and as needed for subsequent treatments. The personal supervision must include participation in a time‐out, assessment of patient immobilization, assessment of adequate imaging parameters, accuracy of respiratory management (if applicable), consultation on excessive or unusual patient shift requirements during treatment not clearly caused by patient motion on the treatment couch, as well as other patient‐ or plan‐specific needs.
