# Supplementary material for: AAPM‐RSS Medical Physics Practice Guideline 9.b: SRS‐SBRT
Source: J Appl Clin Med Phys. 2025 Mar 12;26(4):e14624. doi: 10.1002/acm2.14624 (PMC11969102; doi:10.1002/acm2.14624)

**APPENDIX A: Sample Process Mapping and FMEA**

A full sample of a completed process mapping and FMEA can be found in the WG100 repository <https://mpec.aapm.org/>repository/home.php - Approved Content - Excel template for process mapping and FMEA – Sample completed sheet.

Review of this entire available resource is the best step to understanding the process and initiating an analysis.

**APPENDIX B: SAMPLE Standard Operating Procedure (SOP) DOCUMENT**

(INSTITUTION NAME)

POLICY/PROCEDURE:

STEREOTACTIC BODY RADIATION THERAPY FOR STAGE I PERIPHERAL NON-SMALL CELL LUNG CANCER

POLICY # PHY040

DATES: – START **06-09-12** - BY: **Physicist**

– REVISED **08-27-12**- BY: **Clinical team**

MEDICAL DIRECTOR:  **(MD)**

**POLICY:** This document describes the overall process to be followed when implementing a hypofractionated course of Stereotactic Body Radiation Therapy (SBRT) for peripherally located early stage non-small cell lung cancer. This document describes a process and dose regimen based on RTOG trial 0915. Given the large doses per fraction and the potential for clinically significant complications if a treatment deviates significantly from the recommendations of RTOG 0915, all treatments will be conducted in conformance with the procedure described herein. Unique clinical considerations may require interpretation by the responsible professional (radiation oncologist for clinical matters and medical physicist for technical matters). Ultimately, the judgment of the attending physician must be the controlling factor in the treatment of any specific patient. Nothing herein implies a diminution in such responsibility, nor a trespass upon the physician’s final authority in such matters.

**PROCEDURE:** See attached document.

# (NOTE: This is an EXAMPLE ONLY of a Standard Operating Procedure (SOP) document. The format and scope may, appropriately, vary substantially between institutions and for different clinical applications. The QMP and Medical Director of each clinical program decide on the appropriate scope and format of SOP document(s) to meet their clinical program’s needs.)(Institution Name)

## Physics Procedure dd Month yyyy

SBRT FOR STAGE I PERIPHERAL LUNG CANCER

Qualifications and responsibilities of clinical team members

Each member of the SBRT team must be appropriately trained, and each team member’s responsibilities in the SBRT process must be clearly defined in order to ensure a consistently safe and accurate treatment delivery. We will follow (Institution’s) Physics Policy titled “Qualifications and responsibilities of clinical team members for stereotactic body radiation therapy”, which is based on the recommendations of the ACR-ASTRO Practice Guideline for SBRT^(1)^, ASTRO White Paper on quality and safety considerations in SRS and SBRT^(2)^, and the AAPM Task Group 101 report on SBRT^(3)^.

Patient eligibility

- Medically inoperable, biopsy proven early stage T1, T2 (< 5 cm) NSCLC patients; clinically node negative by PET, with peripherally located tumors (> 2 cm in all directions around the proximal bronchial tree).
- AJCC Stage T1N0M0 or T2N0M0 (<5 cm) as demonstrated by a high-quality diagnostic CT study with intravenous contrast, and a whole-body PET study performed within 8 weeks of simulation.

Exclusion criteria

- T2 tumors >5 cm or involving the central plural and/or structures of the mediastinum.
- The primary tumor of any T-stage within or touching the *zone of the proximal bronchial tree*, defined in RTOG 0915^(4)^ as a volume 2 cm in all directions around the proximal bronchial tree.
- Direct evidence of regional or distant metastases after appropriate staging studies, or synchronous primary malignancy or prior malignancy in the past 2 years except for invasive malignancy that has been treated definitively and the patient remains disease free for > 3 years with life expectancy of > 3 years or carcinoma *in situ* or early stage skin cancers that have been treated definitively.
- Previous radiotherapy to the lung or mediastinum.
- Previous chemotherapy for this lung or mediastinum tumor.
- Previous surgery for this lung or mediastinum tumor.
- Plans for the patient to receive other concomitant therapy (including standard fractionated radiotherapy, chemotherapy, biological therapy, vaccine therapy, and surgery) while on this treatment course except at disease progression.
- Patients with active systemic, pulmonary, or pericardial infection.

### Clinical preparations

All patients who are candidates for the treatment course described herein will receive a Pulmonary Function Test (PFT) to determine baseline performance values, and each patient’s pulmonary function will be evaluated against their baseline values to predict the level of pulmonary toxicity, if any. The following PFT parameters will be recorded: FEV1, FVC, and DLCO. The physician’s consultation will also include an assessment of the patient’s physical and mental condition to determine whether the patient can comply with the requirements of a long treatment session. The informed consent process will include a description of the rationale for hypo-fractionation as well as the alternatives to SBRT.

Patients may receive corticosteroid premedication (e.g., Dexamethasone, 4 mg, p.o. in a single dose, or equivalent) 15-60 minutes before each SBRT treatment for the intended purpose of modulating immediate pulmonary inflammatory effects. Analgesic premedication may also be appropriate, to avoid general discomfort during long treatment sessions**.**

### Patient setup

Patients will be positioned supine, with their head toward the gantry. The “SBRT cradle” (SBRT Pro-Lok by Civco) will be used in combination with one or two vacuum bags, and both arms will be extended above the head. The vacuum bags should be shaped to provide good arm support and hip support to minimize the risk of rotational misalignment. The abdominal compression plate will be used over the upper abdomen / xiphoid process to control the magnitude of respiratory motion; the radiation oncologist will adjust the compression level.

CT simulation

Patients will be CT scanned from approximately the level of the ears to the kidneys, using ≤ 3 mm slice spacing throughout. When appropriate, patients will be scanned using the 4DCT, respiratory correlated imaging technique. At the conclusion of the scanning procedure, the Maximum Intensity Projection (MIP) and Average Intensity studies will be computed. The therapist will export both the average and MIP studies to the planning system.

Contouring

When 4DCT scanning has been performed, we will begin by performing an image fusion of the MIP study to the average study, and will assess the amount of tumor motion based on the MIP study. *If the displacement is 3 mm or greater in any dimension, we will consider the tumor motion to be relevant to planning.* The physician will use the 4D information when determining the GTV.

The following Regions of Interest (ROIs) will be generated [responsible individual in brackets - the physician has final authority/responsibility for ROI definition]:

- **GTV = CTV (no expansion)** [physician]. The GTV should be drawn using a CT pulmonary window, but soft tissue windows may be used to avoid inclusion of adjacent vessels, atelectasis, or mediastinal or chest wall structures within the GTV. 4D information (cine loop or MIP) may be used to incorporate tumor motion into the GTV.
- **PTV** [physician or planner]. Expansion of the GTV by 0.5 cm in all axial directions and 1.0 cm in the craniocaudal direction.
- **Cord** and **Cord+5mm** [planner]. Contour the spinal canal along the length of the lungs, minimum 10 cm beyond the PTV in cranial and caudal directions. Expand 5mm axially for “Cord+5mm”.
- **R lung, L Lung, and Net Lungs** [planner]. Net Lungs is the combination of R and L Lung, subtracting any overlap with the GTV.
- **Heart/pericardium** [planner/physician]. Heart and pericardial sac, starting at the inferior aspect of the aorto-pulmonary window.
- **Esophagus** [planner]. Contoured along the length of the lungs, to the GE junction.
- **Brachial plexus** [physician or planner]. Contour the major trunks of the brachial plexus using the subclavian and axillary vessels as a surrogate for identifying the location of the brachial plexus. This neurovascular complex will be contoured starting proximally at the bifurcation of the brachiocephalic trunk into the jugular/subclavian veins (or carotid/subclavian arteries) and following along the route of the subclavian vein to the axillary vein ending after the neurovascular structures cross the second rib.
- **Stomach** [physician or planner].
- **Ribs** [planner]. Contour all ribs within 5 cm of the PTV.
- **Skin** [planner]. A 0.5cm “rind” from the skin.
- **Great vessels** [physician or planner].
- **Trachea & large bronchus** [physician or planner]. Contour as two separate structures: **Proximal Trachea** and **Proximal Bronchial Tree**. Contouring of the Proximal Trachea should begin at least 10 cm superior to the extent of the PTV or 5 cm superior to the carina (whichever is more superior) and continue inferiorly to the superior aspect of the proximal bronchial tree. The Proximal Bronchial Tree will include the most inferior 2 cm of distal trachea and the proximal airways on both sides. The following airways will be included according to standard anatomic relationships: the distal 2 cm of trachea, the carina, the right and left mainstem bronchi, the right and left upper lobe bronchi, the intermedius bronchus, the right middle lobe bronchus, the lingular bronchus, and the right and left lower lobe bronchi. Contouring of the lobar bronchi will end immediately at the site of a segmental bifurcation.
- **PTV+2cm** [planner]. Expansion of the PTV by 2.0 cm in all directions, minus the PTV.
- **Proximal Bronchial Tree +2cm** [planner]. Expansion of the Proximal Bronchial Tree by 2.0 cm in all directions.

Prospective physician peer review

When the aforementioned contours have been delineated, the attending physician will arrange for a prospective review by another radiation oncologist of the patient eligibility criteria, potential comorbidities, GTV delineation and normal tissue volumes. **The dosimetric treatment planning will not commence until this review has been completed and the attending physician has notified the treatment planning team** through the established communication mechanism (e.g., Quality Checklist in Mosaiq EMR).

### Beam technique

The standard treatment modality will be 6-10MV photons. When necessary, a “mix” of 6/10 MV and higher-energy photons will be used to reduce subcutaneous doses; in such cases, the effect of the high-energy photons on penumbra and secondary buildup at lung-tissue interfaces near the target will be assessed. Every effort will be made to avoid the use of high-energy photon beams that traverse a significant path length of lung tissue before entering the target. The dose distribution should be carefully assessed to minimize the volume of normal lung and heart irradiated, the length of esophagus irradiated, the maximum dose in and near the spinal cord, the subcutaneous dose near skin,

Generally, 10 or more fixed-angle treatment fields, or multiple dynamic arcs if the technology is available, are used for the treatment. The majority of fields should be non-opposing; non-coplanar techniques may be necessary to achieve the desired rapid dose fall-off outside the PTV. Due to the uncertainties related to very-small-field dosimetry for MLC or collimator-jaw shaped fields, a minimum field aperture of 3.5 cm will be enforced.

In order to obtain acceptable target coverage, field aperture size and shape should correspond closely to the projection of the PTV along a beam’s eye view (i.e., the “margin” for dose buildup at the edges of the MLC or collimator jaws beyond the PTV should not exceed 5 mm). The only exception should be when observing the minimum field dimension of 3.5 cm when treating small lesions.

Dosimetric objectives

Dose calculation: While the Adaptive Convolve algorithm and default dose-grid resolution may be used during preliminary treatment plan optimization, the final dose calculation will be performed using the Collapsed Cone Convolution algorithm and a dose-grid resolution of ≤ 0.3 cm. For PTV volumes ≤ 10.0 cc, use 0.2 cm dose-grid resolution.

Prescription dose: The standard prescription will be 12.0 Gy per fraction times 4 fractions for a total dose of 48.0 Gy to the prescription line at the edge of the PTV. The time between fractions is at the discretion of the radiation oncologist, but a minimum of 18 hours is required**.**

Target dose coverage and conformality:

Successful treatment planning will require accomplishment of **all** of the following criteria:

1. **Maximum dose:** The treatment plan should be created such that 100% corresponds to the maximum dose delivered to the patient. This point must exist within the PTV.
2. **Prescription isodose:** The prescription isodose surface must be ≥ 60% and < 90% of the maximum dose.
3. **Prescription isodose surface coverage:** The prescription isodose surface will be chosen such that 95% of the target volume (PTV) is conformally covered by the prescription isodose surface (PTV V95%RX = 100%) and 99% of the target volume (PTV) receives a minimum of 90% of the prescription dose (PTV V90%RX > 99%).
4. **High dose spillage**: The cumulative volume of all tissue outside the PTV receiving a dose > 105% of prescription dose should be no more than 15% of the PTV volume. Conformality of PTV coverage will be judged such that the ratio of the volume of the prescription isodose meeting criteria 1 through 4 to the volume of the PTV (*“conformality index”*) is ideally < 1.2. These criteria will not be required to be met in treating very small tumors (< 2.5 cm axial GTV dimension or < 1.5 cm craniocaudal GTV dimension) in which the required minimum field size of 3.5 cm results in the inability to meet a conformality index of 1.2.

The fall-off gradient beyond the PTV extending into normal tissue structures must be rapid in all directions and meet the following criteria:

1. *Location:* The maximum total dose over all fractions in Gray (Gy) to any point 2 cm or greater away from the PTV in any direction must be no greater than D_2cm_ where D_2cm_ is given by the table below.
2. *Volume:* The ratio of the isodose volume representing 50% of the prescription dose to the volume of the PTV must be no greater than R_50%_ where R_50%_ is given by the table below.

Dose conformality goals:

| PTV volume (cc) | R_50%_ | D_2cm_ (% of Rx) |
| --- | --- | --- |
| 1.8 | 5.9 | 50 |
| 3.8 | 5.5 | 50 |
| 7.4 | 5.1 | 50 |
| 13.2 | 4.7 | 50 |
| 22.0 | 4.5 | 54 |
| 34.0 | 4.3 | 58 |
| 50.0 | 4.0 | 62 |
| 70.0 | 3.5 | 66 |
| 95.0 | 3.3 | 70 |
| 126.0 | 3.1 | 73 |
| 163.0 | 2.9 | 77 |

Normal tissue dose constraints

| Tissue | Volume (cc) | Max dose (Gy) | Max point dose (Gy) |
| --- | --- | --- | --- |
| Spinal cord | 0.35 | 20.8 | 26.0 |
|  | 1.2 | 13.6 |  |
| Esophagus  (avoid circumferential irradiation) | 5.0 | 18.8 | 30.0 |
| Heart/pericardium | 15.0 | 28.0 | 34.0 |
| Bilateral net lungs | 10 % | 20.0 |  |
|  | 1500.0 | 11.6 |  |
|  | 1000.0 | 12.4 |  |
| Brachial plexus | 3.0 | 23.6 | 27.2 |
| Trachea & large bronchus  (avoid circumferential irradiation) | 4.0 | 15.6 | 34.8 |
| Great vessels | 10.0 | 43.0 | 49.0 |
| Ribs | 1.0 | 32.0 | 40.0 |
| Skin (0.5 cm) | 10.0 | 33.2 | 36.0 |
| Stomach | 10.0 | 17.6 | 27.2 |

The physician will complete a Treatment Plan Request form, clearly documenting the prescribed dose(s) and fractionation schedule as well as any dosimetric constraints. In addition to the RTOG 0915 constraints^(4)^, the QUANTEC study^(5)^ provides useful information to be considered.

Plan review and pre-treatment QA:

- When the planner has completed a treatment plan and performed the final dose calculation (see above), the radiation oncologist will carefully review the treatment plan in collaboration with the medical physicist and dosimetrist, verifying adherence to all dosimetric objectives and evaluating the treatment technique for any potential concerns not expressed in the written objectives (e.g. dose of concern to unspecified tissue, complexity of treatment technique).
- Upon physician approval, the dosimetrist will document the chosen treatment technique and export all relevant delivery parameters to the electronic medical record (EMR) and image guidance system, ensuring that all aspects of the chosen treatment technique are clearly conveyed to the therapist team. For unusual or complex aspects of a patient’s treatment technique, communicate directly with the therapists to ensure that the therapist team is aware.
- All isodoses will be displayed in Absolute Dose mode.
- A consistent beam numbering and isocenter labeling method will be followed to clearly identify each treatment isocenter. Capital letters (A,B,C) will be used to designate different isocenters. The label for each “point” will therefore be in the format “A-anatomy”, “B-anatomy” and “C-anatomy” where “anatomy” is a brief anatomic description of the location of the isocenter point (include laterality). Field numbers will be in the format “A1”, “B1” etc. Any treatment plan revisions during the treatment course (extremely rare) will have the usual suffix to denote a revised field, e.g. “A1A”, “B1A” etc.
- The medical physicist will review the final treatment plan for accuracy and deliverability, consulting with the radiation oncologist to ensure that both professionals are confident of the acceptability of the chosen treatment plan.
- The medical physicist will validate the chosen treatment delivery parameters via an independent dose calculation and a phantom measurement. An absolute-dose measurement of the composite dose will be performed in solid water using a micro-chamber. Each beam aperture will be independently measured and compared to the planned aperture, with agreement within 2 mm considered acceptable.
- All physics checks should be completed no later than 24 hours prior to the patient’s first treatment session.
- A therapist who will be present for the first treatment session will complete a pre-treatment review of the chart no later than 12 hours prior to the first treatment session, with particular focus on patient setup instructions, prescribed dose and calculated dose.
- No later than 6 hours prior to the first treatment session, the medical physicist will confer with the therapist team to verify readiness for treatment initiation and to answer any questions regarding the treatment technique.
- The aforementioned steps will be documented through the relevant SBRT “Assessment” tool in the Mosaiq EMR.

If any of the aforementioned pre-treatment checks fail, or are not completed within the specified timeline, the patient’s first treatment session will be postponed and will not be rescheduled until the deficiency has been resolved.

Treatment delivery:

FOR EACH ISOCENTER IN SEQUENCE (A,B,C), follow these steps:

1. The patient will be positioned in the “SBRT cradle” following the set-up instructions recorded at the time of simulation. The radiation oncologist will be present during patient positioning and will adjust the compression level of the abdominal compression plate.
2. The patient will be localized to the reference point using laser alignment and then an in room simulation of all beam angles will be performed to determine clearance and treatment beam order. The mid-point beam will be determined and designated for the mid-treatment position verification (Step 6).
3. Once the patient has been localized to the reference point based on laser alignment and SSD / couch vertical checks, a Cone-Beam CT scan will be acquired and a 3D localization of the reference point will be performed^(6)^. The radiation oncologist and medical physicist will review and approve the resulting alignment. The tolerance for alignment will be 0.3 cm in all dimensions.
4. Once the reference point has been accurately localized, shifts (if any) will be applied to localize the treatment isocenter. Orthogonal portal images will be acquired to verify correct treatment isocenter alignment, with a tolerance for alignment of 0.3 cm.
5. At the first treatment session, one treatment field will be imaged using a double-exposure technique with a generous “delta” for the open exposure. The entire treatment team will review the portal image against the reference image to confirm that the correct isocenter is ready for treatment.
6. [In the early phase of the clinical service, patients may be asked to come for a “dry run session” one day prior to the first actual treatment session, wherein steps 1-4 are completed but no treatment is delivered.]
7. Treatment will be initiated following the [Institution] supervision policy “Qualifications and responsibilities of clinical team members for stereotactic body radiation therapy”.
8. At approximately the mid-point of the treatment session, a second pair of orthogonal portal images will be acquired to verify continued target alignment. If any dimension is misaligned by > 0.5 cm, shifts will be implemented and confirmatory orthogonal portal images acquired. If the nature of the misalignment is ambiguous, a second CBCT scan at the reference point may be necessary, followed by step #3 above.
9. At the completion of the last treatment field for each isocenter, a final pair of orthogonal images will be acquired for off-line data analysis.

REPEAT STEPS 1-8 FOR ADDITIONAL ISOCENTERS.

If at any point during the treatment session there is evidence of significant patient motion (e.g. patient is observed on CCTV monitor to be moving), the treatment will be paused immediately, and an assessment of patient positioning and comfort/compliance will be conducted, followed by target localization as described above.

Patient follow-up:

In addition to the normal on-treatment management, the following scheme will be used for monitoring patients’ outcomes after treatment. In the event that a patient is not able or willing to return for multiple follow-up visits, every effort should be made to ensure that each patient returns for the 6-week and 12-week visits at a minimum.

| Interval post tx | Tests |
| --- | --- |
| 6 weeks | Chest X-ray – evaluate for signs of radiation pneumonitis |
| 12 weeks | PFTs and contrast-enhanced diagnostic CT scan. |
| 1. 6 months | Chest X-ray and PFTs, and FDG-PET scan. |
| 1. 1 year | PFTs and contrast-enhanced diagnostic CT scan |
| Subsequent years | Alternate between (a) and (b) |

Assessment of possible pulmonary toxicity:

We will use the RTOG’s schema^(4)^ for grading pulmonary toxicity, which consists of categories of decline relative to the patient’s baseline values (denoted with a “B” subscript):

| Parameter | Grade 1 | Grade 2 | Grade 3 | Grade 4 |
| --- | --- | --- | --- | --- |
| FEV1 | 0.75 to 0.90 *FEV1_B_ | 0.50 to 0.74 *FEV1_B_ | 0.25 to 0.49 *FEV1_B_ | <0.25 *FEV1_B_ |
| FVC | 0.75 to 0.90 *FVC_B_ | 0.50 to 0.74 *FVC_B_ | 0.25 to 0.49 *FVC_B_ | <0.25 *FVC_B_ |
| DLCO | 0.75 to 0.90 *DLCO_B_ | 0.50 to 0.74 *DLCO_B_ | 0.25 to 0.49 *DLCO_B_ | <0.25 *DLCO_B_ |

Assessment of treatment response:

To assess tumor response to the treatment, we will document the longest diameter (LD) of the GTV from the treatment planning CT scan, and will assess the relative change in this parameter over time. The LD should be measured in all three primary planes (antero-posterior, left-right, and cranio-caudal) from the non-MIP scan (“reference” scan) using a pulmonary CT window, and should be recorded in the clinical assessment section of the patient’s chart for future follow-up. A custom-designed “Assessment” tool in the Mosaiq EMR is recommended for this purpose.

We will use the RTOG’s schema^(4)^ for grading target response, which consists of categories of change relative to the patient’s baseline values (denoted with a “B” subscript):

| Response category | Description |
| --- | --- |
| Complete Response (CR) | Disappearance of the target lesion as determined from CT scan |
| Partial Response (PR) | LD ≤ 0.70 *LD_B_ |
| Stable Disease (SD) | 0.70 *LD_B_ < LD < 1.20 *LD_B_ |
| Local Enlargement (LE) | LD ≥ 1.20 *LD_B_ 🡪 obtain PET scan |
| Local Failure (LF) | LD ≥ 1.20 *LD_B_ **and** PET-avid (similar SUV as baseline PET) |
| Local Control (LC) | The absence of Local Failure |

References

1. “ACR-ASTRO Practice Parameter for the performance of stereotactic body radiation therapy”, American College of Radiology, Reston, VA; 2014.
2. Solberg T, et.al., “Quality and safety considerations in stereotactic radiosurgery and stereotactic body radiation therapy”, Practical Rad Onc Supplement:S1-S49, 2011.
3. Benedict S, et.al., “Stereotactic body radiation therapy: The report of AAPM Task Group 101”, Med Phys 37:4078-4101, 2010. doi:10.1118/1.3438081.
4. Videtic G, Singh A, Chang J, Principal Investigators, “A randomized phase II study comparing 2 stereotactic body radiation therapy (SBRT) schedules for medically inoperable patients with Stage I peripheral non-small cell lung cancer”, Radiation Therapy Oncology Group 0915, Philadelphia PA, 2009, updated 2014.
5. Marks L, et.al., “Quantitative analyses of normal tissue effects in the clinic (QUANTEC),” Int J Rad Onc Bio Phys 76:S1-S160, 2010.
6. Chung H, et.al., “Evaluation of dose variation to normal and critical structures for lung hypofractionated stereotactic body radiation therapy”, Practical Rad Onc 2:e15-e21, 2012.

**APPENDIX C: Sample Process Map for Motion Management**


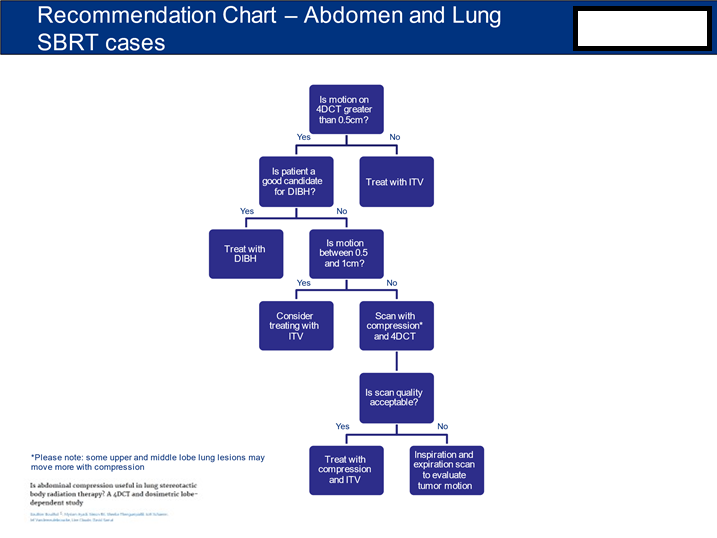

Supplement: Supplementary file 1 — Supporting Information [file ACM2-26-e14624-s001.docx]
